# Targeted metagenomics approach to capture the biodiversity of *Saccharomyces* genus in wild environments

**DOI:** 10.1111/1758-2229.12724

**Published:** 2019-01-13

**Authors:** Haya F. Alsammar, Samina Naseeb, Lorenzo B. Brancia, R. Tucker Gilman, Ping Wang, Daniela Delneri

**Affiliations:** ^1^ Manchester Institute of Biotechnology, Faculty of Biology Medicine and Health The University of Manchester Manchester M1 7DN UK; ^2^ St Ambrose College Altrincham WA15 0HE UK; ^3^ School of Earth and Environmental Sciences, Faculty of Science and Engineering The University of Manchester Manchester M13 9PL UK; ^4^ Genomic Core Facility, Faculty of Biology Medicine and Health The University of Manchester Manchester M13 9PL UK

## Abstract

The species of the genus *Saccharomyces* are commonly inhabiting tree bark and the surrounding soil, but their abundance have likely been underestimated due to biases in culturing methods. Metagenomic studies have so far been unable to detect *Saccharomyces* species in wild environments. Here, we sequenced the mycobiome of soils surrounding different trees at various altitudes in the Italian Alps. To survey for yeasts species belonging to *Saccharomyces* genus rather than other fungal species, we performed a selectivity step involving the isolation of the internal transcribed spacer (ITS) region that is specific to this yeast group. Reads mapping to *Saccharomyces* species were detected in all soil samples, including reads for *S. mikatae* and for *S. eubayanus*. ITS1 alignment of the *S. cerevisiae*, *S. paradoxus* and *S. kudriavzevii* sequences showed up to three base pair polymorphisms with other known strains, indicating possible new lineages. Basidiomycetous fungi were still the dominant species, compared to the Ascomycota, but the selectivity step allowed for the first time the detection and study of the biodiversity of the *Saccharomyces* species in their natural environment.

## Introduction

The *Saccharomyces* species are present in a variety of natural environments, including tree bark, soil, fruits and insects guts (Naumov *et al*., 1998; Sniegowski *et al*., 2002; Stefanini *et al*., 2012; Hyma and Fay, 2013; Charron *et al*., 2014). Although, humans have exploited these species for fermentation and biotechnological purposes for hundreds of years, their ecology, biodiversity and geographic distribution have only recently received attention from the science community. In particular, three new species (*S. arboricola*, *S. eubayanus* and *S. jurei*) have been isolated in the last 10 years, bringing the number of species in the *Saccharomyces* group to eight (Wang and Bai, 2008; Libkind *et al*., 2011; Naseeb *et al*., 2017). Despite the strong association of *S. cerevisiae* with domestication, wild species have been detected at a low frequency in substrates that are isolated from human activity; these wild species have formed geographically structured lineages that are distinct from the domesticated species (Peter *et al*., 2018). The relatively broad ecology of these species occurring in low numbers suggests that they may be generalist nomads (Goddard and Greig, 2015). *S. paradoxus* is a wild species that is frequently associated with oak trees (*Quercus* spp), commonly residing in sympatry with *S. cerevisiae*. The lineages of *S. paradoxus* are differentiated by geographic distance, making this species a model for the study of the natural habitat and evolution of the wild *Saccharomyces* species (Naumov *et al*., 1998; Sniegowski *et al*., 2002; Liti *et al*., 2009).

Far East Asia harbours the broadest diversity of *Saccharomyces* species. *S. kudriavzevii*, *S. mikatae* and *S. arboricola* were initially isolated in this region (along with genetically diverged populations of *S. cerevisiae* and *S. eubayanus*) from soil, decayed leaves and oak bark. (Naumov *et al*., 2000; Wang and Bai, 2008; Bing *et al*., 2014; Peris *et al*., 2014). The distribution of *S. kudriavzevii* and *S. arboricola* has extended out of Far East Asia. *S. kudriavzevii* strains have been detected in Europe (Portugal and Spain), and *S. arboricola* have been reported in Australasia (New Zealand) (Sampaio and Goncalves, 2008; Lopes *et al*., 2010; Gayevskiy and Goddard, 2016). Meanwhile, *S. mikatae* presence is restricted to Far East Asia, and it has not been isolated in any other region. *S. eubayanus* is well‐established in South America. Initially isolated from Southern beech trees (*Nothofagus* spp) in Patagonia, Argentina, *S. eubayanus* has been described as the cryotolerant parent of the lager hybrid *S. pastorianus* (*S. cerevisiae* × *S. eubayanus*) (Libkind *et al*., 2011). The lager yeast was thought to be of Patagonian or Chinese origin, as *S. eubayanus* strains isolated from both regions were highly similar to the non‐*S. cerevisiae* subgenome of *S. pastorianus* (Libkind *et al*., 2011; Bing *et al*., 2014). Population genomic studies of *S. eubayanus* and lager strains have shown that the cryotolerant parent is derived from a lineage of Holarctic distribution; however, none of the analysed wild *S .eubayanus* isolates have been identified as the sole closest relative of the lager yeast (Peris *et al*., 2016). Most wild *S. uvarum* have been isolated from South America coexisting with *S. eubayanus* (Libkind *et al*., 2011; Rodriguez *et al*., 2014). The *S. uvarum* and *S. eubayanus* South American populations are of high genetic diversity, suggesting that these species are native to the region (Almeida *et al*., 2014; Peris *et al*., 2016). *S. jurei* is the latest addition to the *Saccharomyces* species, isolated from oak bark and soil in France (Naseeb *et al*., 2017). Phylogenetic analysis based on 101 concatenated genes has grouped the *S. jurei* strains in the same clade as *S. mikatae*. The two species share a reciprocal chromosomal translocation, suggesting a common evolutionary history (Naseeb *et al*., 2018).

Early studies that sought to isolate *Saccharomyces* species from oak tree bark, surrounding soil and exudates frequently found *S. paradoxus* and *S. cerevisiae*, suggesting that oaks may be the natural habitat of the *Saccharomyces* species (Naumov *et al*., 1998; Sniegowski *et al*., 2002). However, *Saccharomyces* species were also isolated from other tree species, encouraging researchers to explore different habitats (Sampaio and Goncalves, 2008; Bing *et al*., 2014; Charron *et al*., 2014; Sylvester *et al*., 2015). The current understanding of the natural habitats of the *Saccharomyces* species relies on specific enrichment culturing methods. Due to differences in the temperature growth profiles of the species, incubation of samples at varying temperatures have allowed researchers to retrieve a wider range of species. For example, the cryotolerant *S. kudriavzevii* and *S. uvarum* were isolated at 10 °C (Sampaio and Goncalves, 2008). However, substrate enrichment methods inevitably underestimate the variety of wild species, as competition in the enrichment culture reduces the propagation of some species.

To avoid culturing biases, high‐throughput sequencing platforms have been used to study fungal communities existing in natural substrates (Buee *et al*., 2009; Pinto *et al*., 2014; Taylor *et al*., 2014; Masinova *et al*., 2017). Kowallik *et al*. (2015) did not obtain sequences of *Saccharomyces* species from oak bark using high‐throughput sequencing of environmental DNA (eDNA), even though they isolated *S. paradoxus* colonies from the same trees by selective plating (Kowallik *et al*., 2015). A more recent metagenomic study that analysed the yeast community changes in soil and litter affected by biotic and abiotic factors found differences in the basidiomycetes and ascomycetes yeasts among beech (*Fagus* spp), oak and spruce (*Picea* spp) trees, but again, species belonging to the *Saccharomyces* genus were not detected (Masinova *et al*., 2017). Pyrosequencing of eDNA from grapes in New Zealand vineyards has detected *Saccharomyces* species, but in very low numbers (~1:20 000 of sequences) (Taylor *et al*., 2014). A higher number of *S. cerevisiae* and *S. paradoxus* sequences were obtained from DNA extracted from grape must, but fewer sequences for these species were found in the eDNA from oak bark and soil (Dashko *et al*., 2016).

To date, metagenomic studies have eitheir not detected *Saccharomyces* species in natural environments (i.e., contexts not associated with human activities), or they have detected the sequences of a few species in low numbers in substrates related to fermentation activities. However, *Saccharomyces* sequences were recently detected in human‐related samples in relatively high abundance (Boix‐Amoros *et al*., 2017; Nash *et al*., 2017). These results indicate that wild *Saccharomyces* species are in low abundance relative to other fungi and yeasts, hampering the understanding of the species' biodiversity.

The sequencing of eDNA has not previously been used to specifically target *Saccharomyces* species in soil. Here, to capture the diversity of the *Saccharomyces* species associated with the soil surrounding trees at various altitudes, we employed a selection method based on the specific length of the ITS sequence (~850 bp) of the *Saccharomyces* species. Using this technique, we were able to enrich eDNA samples for this yeast group prior to sequencing (using Illumina MiSeq). We detected the molecular signals for *S. mikatae* and *S. eubayanus*, two species that have not previously been isolated in Europe. This is the first study to use an enrichment step specific for the *Saccharomyces* genus, prior to deep eDNA sequencing, to assess the biodiversity of these wild species in areas that are remote from domestication.

## Results and discussion

### *Sequences analysis and OTU clustering*

To have an overview of *Saccharomyces* species in their natural habitats, we have exploited the specific ITS region size of these species (Supporting Information Fig. S2) to limit the interference of other fungi, followed by high‐throughput sequencing of the ITS1 using Illumina MiSeq platform. A total of 27 samples comprising three soil replicates from each of nine trees in three patches at different altitudes (i.e., 600 m, 1400 m and 1800–1900 m) were sequenced (Supporting Information Table S1). After the removal of low quality, chimeric and singleton sequences, 13 088 931 sequences of high quality were obtained from the dataset and clustered into a total of 5578 OTUs. The sequences of the three biological replicates were then grouped to represent the fungal population in the soil of different trees (Supporting Information Table S2). Rarefaction analysis of OTUs in each soil sample (Supporting Information Fig. S3) showed an increase in the number of OTUs with an increase in a number of sequences, with all curves approaching saturation, suggesting that OTU richness was captured. Out of these 5578 OTUs, 1720 OTUs belong to Ascomycetes, 1200 OTUs to Basidiomycetes and the remaining 2658 OTUs were assigned to unclassified read (unidentified), reads with no identity in the databases, or to different phyla (Supporting Information Table S3). Although, we targeted a specific section of the soil mycobiome to select for hemiascomycetes (i.e., ITS corresponding to a region around 850 bp), which constituted 17% of total reads of all samples, a high proportion of OTUs, accounting for 31% of all reads, were assigned to the phylum basidiomycota (Supporting Information Table S3). The average ITS region size of Basidiomycota species is ∼600 bp (Porter and Golding, 2011), it is likely that lower size ITS DNA was still present in the gel extracted area corresponding to the *Saccharomyces* species. Another possibility for the abundance of basidiomycetes is PCR bias towards amplification of shorter length ITS and ITS1 regions (∼600 bp and 214 bp respectively) of basidiomycetes (Porter and Golding, 2011), in contrast to relatively longer regions in *Saccharomyces* species (∼850 bp and 360 bp respectively) (Naumov *et al*., 2000). Moreover, PCR primers were reported to be biased towards the amplification of basidiomycetes (primers ITS1, ITS1‐F and ITS5) or ascomycetes (primers ITS2, ITS3 and ITS4) (Bellemain *et al*., 2010), however, we have reduced this biases by a different combination of primers sequencing in both directions. The amplification bias to a specific *Saccharomyces* species is unlikely, due to the similarity of the species in the size of ITS and ITS1 (Naumov *et al*., 2000). A high number of saprotrophic and mycorrhizal fungi fruiting bodies were visually observed in the sampling locations, especially at areas of 1600 m–1800 m altitude, therefore, accounting for the high proportion of basidiomycetes reads in the eDNA. Surveys of fungal diversity in the soil of forests populated with a variety of trees using pyrosequencing revealed the similar dominance of Basidiomycota fungi (Buee *et al*., 2009).

Our data included samples from both beech and spruce trees at 600 m and 1400 m and no other tree species were sampled at more than one location. Focusing on beech and spruce samples, we found that fungal communities (represented as OTUs) detected in the same location from different tree species were more similar than those collected at different locations from the same tree species (Table 1). This suggests that, even at a moderate spatial scale, location plays a more important role in determining the fungal community than tree species.

**Table 1:** Jaccard similarity coefficients for fungal communities collected from different tree species at the same location (rows 1 and 2), and for the same tree species at different locations (rows 3 and 4).

Trees in comparison	Jaccard similarity coefficient
Spruce, 600 m versus beech, 600 m	0.291
Spruce, 1400 m versus beech, 1400 m	0.288
Spruce, 600 m versus spruce 1400 m	0.213
Beech, 600 m versus beech 1400 m	0.190

Higher similarity coefficients indicate more similar communities.

### *The diversity of Saccharomyces species in soil surrounding trees at varying altitudes*

**Figure 1:**
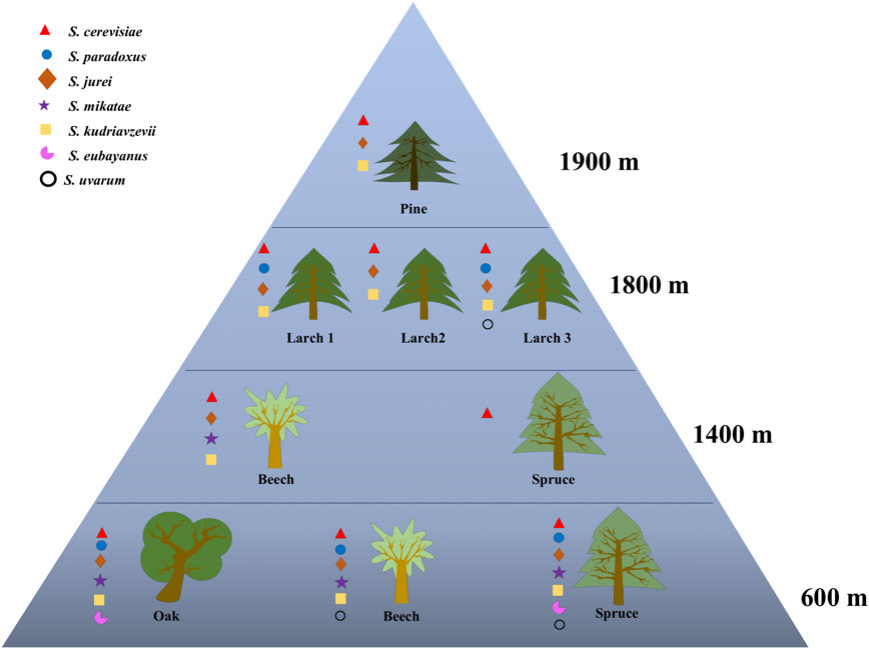
Distribution of *Saccharomyces* species in the mycobiome of soil surrounding different tree species at elevated altitudes. The presence of species is expressed as Operational taxonomic units (OTUs) which consist of at least two identical reads. Three individual Larch trees were sampled in the area of 1800 m altitude.

Sequencing of the gel‐extracted ITS1 succeeded in detecting *S. kudriavzevii*, *S. mikatae*, *S. cerevisiae*, *S. paradoxus*, *S. eubayanus, S. jurei* and *S. uvarum* in our samples, representing about 0.1% of all Ascomycota sequences, therefore, these species are rare in wild environments relative to other fungal species (Fig. 1). The identity of *Saccharomyces* species obtained using QIIME 12_11 ITS database was manually confirmed by comparison to NCBI database using Blast. Most *Saccharomyces* species reads showed 99% similarity to the corresponding NCBI GenBank reference, with 100% sequence coverage (Table 2). The taxonomy of three *Saccharomyces* species reads assigned by QIIME 12_11 database were not the same as the GenBank reference. OTU 1152 was assigned to *S. bayanus*; however, the sequences corresponded to ‘uncultured fungus clone’. Alignment of reads assigned as *S. spencerorum* (OTU 1159, Supporting Information Table S2), a species that have been renamed to the genus *Kazachstania* as *K. spencerorum* (Kurtzman and Robnett, 2003), to GenBank references resulted in 99% similarity to *S. jurei* NCYC 3947^T^. OTU 1159 aligned to the ITS1 region of *S. jurei* NCYC 3947^T^, however, did not align to the ITS1 of *K. spencerorum* (Supporting Information Fig. S4). Reads of OTU 1158 were assigned as *S. uvarum* instead of *S. pastorianus* as the sequences were identified as *S. uvarum* against the GenBank database. Moreover, the sequence of OTU 1158 showed similarity to ITS1 of *S. uvarum* NRRL Y‐17034^T^ in the two regions that differentiate *S. uvarum* from *S. pastorianus* NRRL Y‐2717N^T^ and *S. bayanus* NRRL Y‐12624^T^ (Supporting Information Fig. S5). QIIME 12_11 can misidentify some *Saccharomyces* species reads due to errors in taxonomy assignment in the database and lack of recent updates of ITS sequences databases (Vilgalys, 2003). We were able to overcome the poor taxonomic annotation of the ITS1 sequences deposited in the QIIME 12_11 database by the alignment of the individual *Saccharomyces* sequences to the reference sequences of the NCBI database. Previous attempts to study the diversity of *Saccharomyces* species in oak trees bark in nature through pyrosequencing did not obtain any reads corresponding to *Saccharomyces* (Kowallik *et al*., 2015). This indicates that the selectivity step used in this study is effective in capturing sequences of most *Saccharomyces* species present in nature.

**Table 2:** Operational taxonomic units of *Saccharomyces* species in the dataset aligned to reference sequences of NCBI database.

OTU	Species (Accession number)	Similarity to reference species (%)	Coverage (%)
OTU 1153	*S. cerevisiae* (MG241531)	99	100
OTU 1157	*S. paradoxus* (MH032820)	99	99
OTU 1156	*S. mikatae* (KY105203)	99	100
OTU 1159	*S. jurei* (HG764814)	99	100
OTU 1155	*S. kudriavzevii* (CP030973)	99	100
OTU 1154	*S. eubayanus* (CP030956)	100	100
OTU 1158	*S. uvarum* (MH459413)	99	100

Sequences of *S. eubayanus* were found in the soil of oak and spruce at 600 m (Fig. 1). This study presents the first evidence of *S. eubayanus* presence in Europe based on sequencing of the mycobiome. Extensive sampling of different tree species in South America and China resulted in the isolation of a population of *S. uvarum* existing in sympatry with *S. eubayanus* (Libkind *et al*., 2011; Bing *et al*., 2014). In our data, *S. uvarum* (detected as *S. pastorianus* QIIME 12_11 ITS database and manually corrected) co‐occurred with *S. eubayanus* in soil from spruce at 600 m. *S. uvarum* was also detected in soil from a beech tree at 600 m and larch 3 at 1800 m (Fig. 1).

*S. kudriavzevii* and *S. jurei* were each present in eight out of nine trees sampled (*S. jurei* and *S. kudriavzevii* were absent from spruce soil at 1400 m), while *S. cerevisiae* was in the soil of all trees. Interestingly, *S. paradoxus*, which is the *Saccharomyces* species most commonly isolated from natural environments (Naumov *et al*., 1998; Sniegowski *et al*., 2002; Sampaio and Goncalves, 2008), was detected in only five out of nine trees.

In agreement with previous results on the coexistence of *S. cerevisiae* and *S. paradoxus*, both species were recorded in the soil of trees at 600 m and two trees at 1800 m (Fig. 1) (Naumov *et al*., 1998; Sniegowski *et al*., 2002; Sweeney *et al*., 2004). Although, *S. cerevisiae* has been thoroughly domesticated, wild strains forming distinct populations have been isolated from natural substrates such as soil, bark and tree exudates (Naumov *et al*., 1998; Sniegowski *et al*., 2002; Fay and Benavides, 2005; Wang *et al*., 2012; Almeida *et al*., 2015). Phylogenetic analysis based on single nucleotide polymorphisms (SNPs) of a large number of *S. cerevisiae* from different habitats and geographical regions revealed domesticated population and wild population as two separated lineages separated by mosaic strains of admixture lineages that are human‐related (Peter *et al*., 2018). However, previous metagenomics studies on fungal diversity in soil and bark of different tree species have failed to detect *S. cerevisiae* (Buee *et al*., 2009; Cordier *et al*., 2012; Kowallik *et al*., 2015; Masinova *et al*., 2017), probably due to the higher number of reads corresponding to the more abundant fungi in these areas. The latest addition to the *Saccharomyces* species, *S. jurei,* had been isolated in the St Auban region of France (Naseeb *et al*., 2017). Our results show that *S. jurei* is also present in other pre‐alpine and alpine environments.

*S. mikatae* reads were detected in soil samples of all trees at 600 m and in the soil of beech at 1400 m. Sequences of this species were detected recently in grape must from European vineyards in Slovenia (Dashko *et al*., 2016). Our study is the first to demonstrate the presence of wild *S. mikatae* in areas without domestication activities.

The soil of oak tree harboured over twofold the number of *Saccharomyces* species reads in comparison to samples of different trees at the same altitude (Table 3), which may indicate the oak tree as the preferred habitat of these species. Despite the isolation of *Saccharomyces* species from different tree species, (Libkind *et al*., 2011; Bing *et al*., 2014; Rodriguez *et al*., 2014; Gayevskiy and Goddard, 2016), most *Saccharomyces* species show a relatively strong association with oak tree, especially in the Northern Hemisphere (Sniegowski *et al*., 2002; Sampaio and Goncalves, 2008; Charron *et al*., 2014; Sylvester *et al*., 2015).

**Table 3:** Number of *Saccharomyces* reads in soil samples surrounding trees at varying altitudes.

Tree, altitude	*S. cerevisiae*	*S. paradoxus*	*S. jurei*	*S. mikatae*	*S. kudriavzevii*	*S. eubayanus*	*S. uvarum*
Oak 600 m	24	18	24	132	316	1	0
Beech 600 m	85	7	23	41	50	0	9
Spruce 600 m	21	6	13	29	42	2	2
Beech 1400 m	13	0	3	27	183	0	0
Spruce 1400 m	2	0	0	0	0	0	0
Larch 1 1800 m	2	2	2	0	7	0	0
Larch 2 1800 m	3	0	65	0	158	0	0
Larch 3 1800 m	6	10	2	0	44	0	3
Pine 1900 m	1	0	1	0	8	0	0

**Figure 2:**
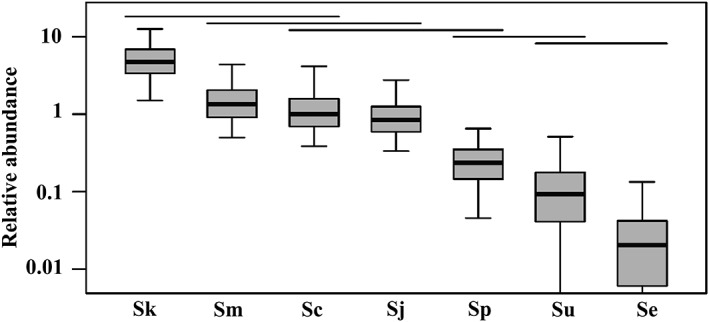
Relative abundance of seven *Saccharomyces* species across all samples in our study. *Saccharomyces* species are abbreviated as: Sk (*S. kudriavzevii*), Sm (*S. mikatae*), Sj (*S. jurei*), Sc (*S. cerevisiae*), Sp (*S. paradoxus*), Su (*S.uvarum*) and Se (*S. eubayanus*). Abundance is reported relative to the abundance of *S. cerevisiae* in the observed data. Box plots show 50% (boxes) and 95% (whiskers) confidence intervals around the observed relative abundance. Our bootstrap analysis could not identify a lower bound for the 95% confidence interval around the abundance of *S. uvarum* or *S. eubayanus*. The abundance of species under the same horizontal line are not significantly different (study‐wide type I error rate *α* = 0.05).

The best model for our read count data included effects of species, patch and the interaction between species and patch (likelihood ratio of the second best to the best model, < 10^−10^). *Saccharomyces* species in our samples had different abundances (expressed as number of reads), and the relative abundances of the species differed among patches (Table 3). Across most samples in most patches, *S. kudriavzevii* was more abundant than any other species (Fig. 2, Table 3). The presence of *S. kudriavzevii* in most soil samples coupled with its abundance relative to other *Saccharomyces* species suggests that it is well established in the sampling area (Table 3).

*S. paradoxus* is geographically widely distributed and can be readily isolated in high abundance from tree bark and surrounding soil using enrichment culturing protocols (Naumov *et al*., 1998; Sniegowski *et al*., 2002; Liti *et al*., 2009; Charron *et al*., 2014). However, in our samples, *S. paradoxus* was less abundant than *S. kudriavzevii* or *S. mikatae*. Thus, either *S. paradoxus* was less common in our samples than in previous studies, or the use of enrichment cultures to evaluate the diversity of wild *Saccharomyces* species is biased towards detecting *S. paradoxus*. The latter might be true if *S. paradoxus* is able to outcompete other *Saccharomyces* species in laboratory enrichment cultures. In fact, Kowallik *et al*. (2015) reported the inhibition of *S. paradoxus* growth in its natural habitat caused by the surrounding microbial community. *S. eubayanus* was less abundant in our samples than any other species except possibly *S. uvarum* (Fig. 2). The low amount of reads found for *S. eubayanus* may explain why it has not previously been isolated in Europe.

**Figure 3:**
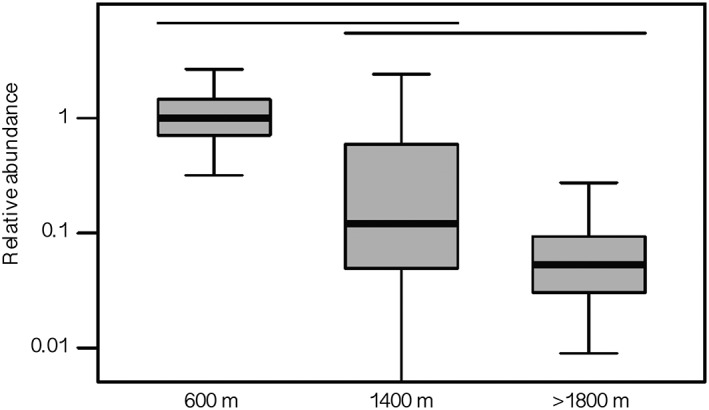
Relative abundance of *Saccharomyces* in patches at three different altitudes. Abundance is reported relative to the abundance of *Saccharomyces* in the patch at 600 m. Box plots show 50% (boxes) and 95% (whiskers) confidence intervals around the observed relative abundance. Our bootstrap analysis could not identify a lower bound for the 95% confidence interval around the abundance of *Saccharomyces* in the patch at 1400 m. Abundance in patches under the same horizontal line are not significantly different (study‐wide type I error rate *α* = 0.05).

*Saccharomyces* species were more abundant in the patch at 600 m than in the patches 1400 m or 1800–1900 m (Fig. 3), indicating a possible consequence of temperature decreases with altitude, which can shape ecological communities (Korner, 2007). We obtained no *S. mikatae* reads, and few *S. cerevisiae* reads from soils collected above 1400 m (Table 3). The optimum growth temperatures of *S. mikatae* and *S. cerevisiae* are approximately 29 °C and 32 °C respectively (Salvado *et al*., 2011). We hypothesise that the thermosensitive nature of these species may have contributed to their reduced abundance in the high‐altitude patch. *S. kudriavzevii* and *S. jurei* appear to be relatively more abundant than other *Saccharomyces* species in soil collected at altitude 1800 m (Table 3). The abundance of the species at high altitudes is correlated with their temperature growth profile, *S. kudriavzevii* is characterized as a cryotolerant species grows optimally at approximately 23 °C and *S. jurei* is able to grow at high and low temperatures with optimum growth temperature higher than *S. kudriavzevii* (∼25°C–30°C) (Salvado *et al*., 2011; Naseeb *et al*., 2018).

### Saccharomyces *reads alignment with genetically distinct populations*

We aligned the ITS1 sequences in our samples with those of known *Saccharomyces* species and found unique base pair differences in *S. cerevisiae*, *S. paradoxus* and *S. kudriavzevii*. This suggests the presence of new distinct Italian lineage of these species.

Diverged populations within wild *Saccharomyces* species have been related to different geographical origins (Liti *et al*., 2009; Wang *et al*., 2012; Almeida *et al*., 2014; Peris *et al*., 2014; Peris *et al*., 2016). Recently, whole‐genome sequencing of over 1000 *S. cerevisiae* strains revealed strains clustering into lineages that are associated with ecological niche, geographical origin and domestication (Peter *et al*., 2018). Liti *et al*. identified five genetically diverged wild and domesticated populations of *S. cerevisiae* (West African, Malaysian, Sake, North American and Wine/European) (Liti *et al*., 2009). Later, eight distinct lineages of wild *S. cerevisiae* were identified from different regions of China (Wang *et al*., 2012). Here, we report a *S. cerevisiae* population in which the OTU 1153 sequence possess three unique base pair difference from previously reported *S. cerevisiae* populations and is closely related to the European strain indicated by an identical nucleotide in the ITS1 sequence that is specific to the strains (Supporting Information Fig. S6).

*S. paradoxus* strains are clustered into four genetically distinct populations correlated with the geographical origin (Liti *et al*., 2009). Alignment of our *S. paradoxus* ITS1 sequence with those from the European, American, Far Eastern and Hawaiian populations shows the segregation of *S. paradoxus* OTU 1157 by three base pairs (Supporting Information Fig. S7), indicating that this is a potentially distinct lineage. However, the ITS1 sequence of OTU 1157 shares the same base pair with the European (CBS 432) and Far Eastern (N 45) populations that separate these species from the American (YPS138) and Hawaiian strains (UWOPS91.917.1).

Natural isolates of *S. kudriavzevii* are distributed into three genetically diverged populations. The European population includes Spanish and Portuguese isolates, a Japanese population consisting of the type strain IFO 1802^T^, and a second Japanese population that includes IFO 1803 that is diverged from IFO 1802^T^ by 4% (Hittinger *et al*., 2010; Lopes *et al*., 2010). The different populations of *S. kudriavzevii* differ in the ITS1 sequence by two nucleotides (Supporting Information Fig. S8) The ITS1 sequence of *S. kudriavzevii* OTU 1155 is more similar to the ITS1 of the Spanish strain CA111 than to the Portuguese strain (ZP591), as indicated by a single common base pair between the strains (Supporting Information Fig. S8). However, the ITS1 sequence of the Italian population differed from all known sequences (i.e., European and Asian strains) by two unique base pair. The presence of unique base pair substitutions in the Italian *S. kudriavzevii* ITS1 sequence again points to a new potential European lineage of *S. kudriavzevii*.

*S. eubayanus* (OTU 1154) ITS1 sequence was not aligned with ITS1 of previously identified populations of *S. eubayanus* as the sequence of this loci is identical in the known populations (Peris *et al*., 2014). The ITS1 sequence of *S. uvarum* differs from *S. eubayanus* by one base pair (Peris *et al*., 2014). This base pair difference was also detected between *S. eubayanus* OTU 1154 and *S. uvarum* NRRL Y‐17034^T^ in our samples (Supporting Information Fig. S9).

Population structure studies are not possible yet for *S. mikatae* and *S. jurei* since *S. mikatae* strains (IFO1815^T^ and IFO 1816) were isolated only in Asia, while *S. jurei* strains (NCYC3947^T^ and NCYC 3962) were only isolated in France (Naumov *et al*., 2000; Naseeb *et al*., 2017). Here, *S. mikatae* OTU 1156 and *S. jurei* OTU 1159 showed one and two base pair substitution, respectively, with their type strains (Table 2).

## Conclusion

We have demonstrated that high‐throughput sequencing of the ITS1 regions amplified from size‐specific ITS is effective in capturing the diversity of wild *Saccharomyces* species in their habitat. This method allowed for the first time the detection of most natural *Saccharomyces* species including *S. eubayanus* and *S. mikatae*, which had not previously been isolated from Europe, and *S. jurei* which is a newly discovered species originally isolated in France. Potential signs of *S. eubayanus* in the Italian Alps will encourage focused field work to isolate this species in Europe where brewing initiated in the 1400s. A general high number of fungal OTUs shared between the same patch rather than the tree type suggests that the fungal soil community depends more strongly on the soil location than the tree type. However, *Saccharomyces* species reads were noticeably more abundant in soil of oak tree than in soil of other trees at the same sampling location, which refers to a possible specificity of *Saccharomyces* species to oak tree. *S. kudriavzevii*, *S. cerevisiae* and *S. paradoxus* sequences of ITS1 show up to three base pair differences from the ITS1 sequences of specific strains belonging to previously described populations, suggesting that they may represent novel Italian lineages.

Our results suggest that culturing methods may have introduced bias into the isolation of some *Saccharomyces* species, since *S. paradoxus*, which has been widely isolated by enrichment culturing, had lower abundance than *S. kudriavzevii* and *S. mikatae* in our data. However, we cannot exclude the possibility that high abundance of *S. kudriavzevii* and *S. mikatae* may be specific to the sampling regions.

Overall, by using a targeted metagenomic approach, we were able for the first time to detect the species belonging to *Saccharomyces* genus in their natural habitat. Moreover, our results suggest the presence of new *Saccharomyces* populations and our method can be applied to systematic sampling to gain a better understanding of the ecology and evolution of *Saccharomyces* species.

## Conflict of interest

The authors declare no conflict of interest.

## Supporting information


**Appendix S1**: Supporting information.

**Table S1.** Location of soil samples collected surrounding different tree species at varying altitudes.
**Table S2.** OTUs count of fungal species across all samples of soil.
**Table S3.** Abundance and diversity of fungal phyla.

## References

[emi412724-bib-0001] Almeida, P. , Goncalves, C. , Teixeira, S. , Libkind, D. , Bontrager, M. , Masneuf‐Pomarede, I. , *et al* (2014) A Gondwanan imprint on global diversity and domestication of wine and cider yeast *Saccharomyces uvarum* . Nat Commun 5: 4044. 24887054 10.1038/ncomms5044 PMC5081218

[emi412724-bib-0002] Almeida, P. , Barbosa, R. , Zalar, P. , Imanishi, Y. , Shimizu, K. , Turchetti, B. , *et al* (2015) A population genomics insight into the Mediterranean origins of wine yeast domestication. Mol Ecol 24: 5412–5427. 26248006 10.1111/mec.13341

[emi412724-bib-0003] Bellemain, E. , Carlsen, T. , Brochmann, C. , Coissac, E. , Taberlet, P. , and Kauserud, H. (2010) ITS as an environmental DNA barcode for fungi: an in silico approach reveals potential PCR biases. BMC Microbiol 10: 189. 20618939 10.1186/1471-2180-10-189 PMC2909996

[emi412724-bib-0004] Bing, J. , Han, P.J. , Liu, W.Q. , Wang, Q.M. , and Bai, F.Y. (2014) Evidence for a Far East Asian origin of lager beer yeast. Curr Biol 24: R380–R381. 24845661 10.1016/j.cub.2014.04.031

[emi412724-bib-0005] Boix‐Amoros, A. , Martinez‐Costa, C. , Querol, A. , Collado, M.C. , and Mira, A. (2017) Multiple approaches detect the presence of fungi in human Breastmilk samples from healthy mothers. Sci Rep 7: 13016. 29026146 10.1038/s41598-017-13270-x PMC5638952

[emi412724-bib-0006] Buee, M. , Reich, M. , Murat, C. , Morin, E. , Nilsson, R.H. , Uroz, S. , and Martin, F. (2009) 454 pyrosequencing analyses of forest soils reveal an unexpectedly high fungal diversity. New Phytol 184: 449–456. 19703112 10.1111/j.1469-8137.2009.03003.x

[emi412724-bib-0007] Charron, G. , Leducq, J.B. , Bertin, C. , Dube, A.K. , and Landry, C.R. (2014) Exploring the northern limit of the distribution of *Saccharomyces cerevisiae* and *Saccharomyces paradoxus* in North America. FEMS Yeast Res 14: 281–288. 24119009 10.1111/1567-1364.12100

[emi412724-bib-0008] Cordier, T. , Robin, C. , Capdevielle, X. , Fabreguettes, O. , Desprez‐Loustau, M.L. , and Vacher, C. (2012) The composition of phyllosphere fungal assemblages of European beech (*Fagus sylvatica*) varies significantly along an elevation gradient. New Phytol 196: 510–519. 22934891 10.1111/j.1469-8137.2012.04284.x

[emi412724-bib-0009] Dashko, S. , Liu, P. , Volk, H. , Butinar, L. , Piskur, J. , and Fay, J.C. (2016) Changes in the relative abundance of two *Saccharomyces* species from oak forests to wine fermentations. Front Microbiol 7: 215. 26941733 10.3389/fmicb.2016.00215 PMC4764737

[emi412724-bib-0010] Fay, J.C. , and Benavides, J.A. (2005) Evidence for domesticated and wild populations of *Saccharomyces cerevisiae* . PLoS Genet 1: 66–71. 16103919 10.1371/journal.pgen.0010005 PMC1183524

[emi412724-bib-0011] Gayevskiy, V. , and Goddard, M.R. (2016) *Saccharomyces eubayanus* and *Saccharomyces arboricola* reside in North Island native New Zealand forests. Environ Microbiol 18: 1137–1147. 26522264 10.1111/1462-2920.13107

[emi412724-bib-0012] Goddard, M.R. , and Greig, D. (2015) *Saccharomyces cerevisiae*: a nomadic yeast with no niche? FEMS Yeast Res 15: 1–6. 10.1093/femsyr/fov009 PMC4444983 25725024

[emi412724-bib-0013] Hittinger, C.T. , Goncalves, P. , Sampaio, J.P. , Dover, J. , Johnston, M. , and Rokas, A. (2010) Remarkably ancient balanced polymorphisms in a multi‐locus gene network. Nature 464: 54–58. 20164837 10.1038/nature08791 PMC2834422

[emi412724-bib-0014] Hyma, K.E. , and Fay, J.C. (2013) Mixing of vineyard and oak‐tree ecotypes of *Saccharomyces cerevisiae* in North American vineyards. Mol Ecol 22: 2917–2930. 23286354 10.1111/mec.12155 PMC3620907

[emi412724-bib-0015] Korner, C. (2007) The use of ‘altitude’ in ecological research. Trends Ecol Evol 22: 569–574. 17988759 10.1016/j.tree.2007.09.006

[emi412724-bib-0016] Kowallik, V. , Miller, E. , and Greig, D. (2015) The interaction of *Saccharomyces paradoxus* with its natural competitors on oak bark. Mol Ecol 24: 1596–1610. 25706044 10.1111/mec.13120 PMC4405091

[emi412724-bib-0017] Kurtzman, C.P. , and Robnett, C.J. (2003) Phylogenetic relationships among yeasts of the *‘Saccharomyces* complex’ determined from multigene sequence analyses. FEMS Yeast Res 3: 417–432. 12748053 10.1016/S1567-1356(03)00012-6

[emi412724-bib-0018] Libkind, D. , Hittinger, C.T. , Valerio, E. , Goncalves, C. , Dover, J. , Johnston, M. , *et al* (2011) Microbe domestication and the identification of the wild genetic stock of lager‐brewing yeast. Proc Natl Acad Sci USA 108: 14539–14544. 21873232 10.1073/pnas.1105430108 PMC3167505

[emi412724-bib-0019] Liti, G. , Carter, D.M. , Moses, A.M. , Warringer, J. , Parts, L. , James, S.A. , *et al* (2009) Population genomics of domestic and wild yeasts. Nature 458: 337–341. 19212322 10.1038/nature07743 PMC2659681

[emi412724-bib-0020] Lopes, C.A. , Barrio, E. , and Querol, A. (2010) Natural hybrids of *S. cerevisiae* x *S. kudriavzevii* share alleles with European wild populations of *Saccharomyces kudriavzevii* . FEMS Yeast Res 10: 412–421. 20337723 10.1111/j.1567-1364.2010.00614.x

[emi412724-bib-0021] Masinova, T. , Bahnmann, B.D. , Vetrovsky, T. , Tomsovsky, M. , Merunkova, K. , and Baldrian, P. (2017) Drivers of yeast community composition in the litter and soil of a temperate forest. FEMS Microbiol Ecol 93: 2. 10.1093/femsec/fiw223 27789535

[emi412724-bib-0022] Naseeb, S. , Alsammar, H. , Burgis, T. , Donaldson, I. , Knyazev, N. , Knight, C. , and Delneri, D. (2018) Whole genome sequencing, de novo assembly and phenotypic profiling for the new budding yeast species *Saccharomyces jurei* . G3: Genes|Genomes|Genetics 8: 2967–2977. 30097472 10.1534/g3.118.200476 PMC6118302

[emi412724-bib-0023] Naseeb, S. , James, S.A. , Alsammar, H. , Michaels, C.J. , Gini, B. , Nueno‐Palop, C. , *et al* (2017) *Saccharomyces jurei* sp. nov., isolation and genetic identification of a novel yeast species from *Quercus robur* . Int J Syst Evol Microbiol 67: 2046–2052. 28639933 10.1099/ijsem.0.002013 PMC5817255

[emi412724-bib-0024] Nash, A.K. , Auchtung, T.A. , Wong, M.C. , Smith, D.P. , Gesell, J.R. , Ross, M.C. , *et al* (2017) The gut mycobiome of the human microbiome project healthy cohort. Microbiome 5: 153. 29178920 10.1186/s40168-017-0373-4 PMC5702186

[emi412724-bib-0025] Naumov, G.I. , Naumova, E.S. , and Sniegowski, P.D. (1998) *Saccharomyces paradoxus* and *Saccharomyces cerevisiae* are associated with exudates of North American oaks. Can J Microbiol 44: 1045–1050. 10029999

[emi412724-bib-0026] Naumov, G.I. , James, S.A. , Naumova, E.S. , Louis, E.J. , and Roberts, I.N. (2000) Three new species in the *Saccharomyces sensu stricto* complex: *Saccharomyces cariocanus*, *Saccharomyces kudriavzevii* and *Saccharomyces mikatae* . Int J Syst Evol Microbiol 50: 1931–1942. 11034507 10.1099/00207713-50-5-1931

[emi412724-bib-0027] Peris, D. , Sylvester, K. , Libkind, D. , Goncalves, P. , Sampaio, J.P. , Alexander, W.G. , and Hittinger, C.T. (2014) Population structure and reticulate evolution of *Saccharomyces eubayanus* and its lager‐brewing hybrids. Mol Ecol 23: 2031–2045. 24612382 10.1111/mec.12702

[emi412724-bib-0028] Peris, D. , Langdon, Q.K. , Moriarty, R.V. , Sylvester, K. , Bontrager, M. , Charron, G. , *et al* (2016) Complex ancestries of lager‐brewing hybrids were shaped by standing variation in the wild yeast *Saccharomyces eubayanus* . PLoS Genet 12: e1006155. 27385107 10.1371/journal.pgen.1006155 PMC4934787

[emi412724-bib-0029] Peter, J. , De Chiara, M. , Friedrich, A. , Yue, J.X. , Pflieger, D. , Bergstrom, A. , *et al* (2018) Genome evolution across 1,011 *Saccharomyces cerevisiae* isolates. Nature 556: 339–344. 29643504 10.1038/s41586-018-0030-5 PMC6784862

[emi412724-bib-0030] Pinto, C. , Pinho, D. , Sousa, S. , Pinheiro, M. , Egas, C. , and Gomes, A.C. (2014) Unravelling the diversity of grapevine microbiome. PLoS One 9: e85622. 24454903 10.1371/journal.pone.0085622 PMC3894198

[emi412724-bib-0031] Porter, T.M. , and Golding, G.B. (2011) Are similarity‐ or phylogeny‐based methods more appropriate for classifying internal transcribed spacer (ITS) metagenomic amplicons? New Phytol 192: 775–782. 21806618 10.1111/j.1469-8137.2011.03838.x

[emi412724-bib-0032] Rodriguez, M.E. , Perez‐Traves, L. , Sangorrin, M.P. , Barrio, E. , and Lopes, C.A. (2014) *Saccharomyces eubayanus* and *Saccharomyces uvarum* associated with the fermentation of *Araucaria araucana* seeds in Patagonia. FEMS Yeast Res 14: 948–965. 25041507 10.1111/1567-1364.12183

[emi412724-bib-0033] Salvado, Z. , Arroyo‐Lopez, F.N. , Guillamon, J.M. , Salazar, G. , Querol, A. , and Barrio, E. (2011) Temperature adaptation markedly determines evolution within the genus *Saccharomyces* . Appl Environ Microbiol 77: 2292–2302. 21317255 10.1128/AEM.01861-10 PMC3067424

[emi412724-bib-0034] Sampaio, J.P. , and Goncalves, P. (2008) Natural populations of *Saccharomyces kudriavzevii* in Portugal are associated with oak bark and are sympatric with *S. cerevisiae* and *S. paradoxus* . Appl Environ Microbiol 74: 2144–2152. 18281431 10.1128/AEM.02396-07 PMC2292605

[emi412724-bib-0035] Sniegowski, P.D. , Dombrowski, P.G. , and Fingerman, E. (2002) *Saccharomyces cerevisiae* and *Saccharomyces paradoxus* coexist in a natural woodland site in North America and display different levels of reproductive isolation from European conspecifics. FEMS Yeast Res 1: 299–306. 12702333 10.1111/j.1567-1364.2002.tb00048.x

[emi412724-bib-0036] Stefanini, I. , Dapporto, L. , Legras, J.L. , Calabretta, A. , Di Paola, M. , De Filippo, C. , *et al* (2012) Role of social wasps in *Saccharomyces cerevisiae* ecology and evolution. Proc Natl Acad Sci USA 109: 13398–13403. 22847440 10.1073/pnas.1208362109 PMC3421210

[emi412724-bib-0037] Sweeney, J.Y. , Kuehne, H.A. , and Sniegowski, P.D. (2004) Sympatric natural *Saccharomyces cerevisiae* and *S. paradoxus* populations have different thermal growth profiles. FEMS Yeast Res 4: 521–525. 14734033 10.1016/S1567-1356(03)00171-5

[emi412724-bib-0038] Sylvester, K. , Wang, Q.M. , James, B. , Mendez, R. , Hulfachor, A.B. , and Hittinger, C.T. (2015) Temperature and host preferences drive the diversification of *Saccharomyces* and other yeasts: a survey and the discovery of eight new yeast species. FEMS Yeast Res 15: 1–16. 10.1093/femsyr/fov002 25743785

[emi412724-bib-0039] Taylor, M.W. , Tsai, P. , Anfang, N. , Ross, H.A. , and Goddard, M.R. (2014) Pyrosequencing reveals regional differences in fruit‐associated fungal communities. Environ Microbiol 16: 2848–2858. 24650123 10.1111/1462-2920.12456 PMC4257574

[emi412724-bib-7040] Vilgalys, R. (2003) Taxonomic misidentification in public DNA databases. New Phytologist 160: 4–5. 10.1046/j.1469-8137.2003.00894.x 33873532

[emi412724-bib-0040] Wang, Q.M. , Liu, W.Q. , Liti, G. , Wang, S.A. , and Bai, F.Y. (2012) Surprisingly diverged populations of *Saccharomyces cerevisiae* in natural environments remote from human activity. Mol Ecol 21: 5404–5417. 22913817 10.1111/j.1365-294X.2012.05732.x

[emi412724-bib-0041] Wang, S.A. , and Bai, F.Y. (2008) *Saccharomyces arboricolus* sp. nov., a yeast species from tree bark. Int J Syst Evol Microbiol 58: 510–514. 18218959 10.1099/ijs.0.65331-0

