# Supplementary material for: Targeted metagenomics approach to capture the biodiversity of Saccharomyces genus in wild environments
Source: Environ Microbiol Rep. 2019 Jan 13;11(2):206–14. doi: 10.1111/1758-2229.12724 (PMC6767435; doi:10.1111/1758-2229.12724)
Supplement: Supplementary file 1 — Appendix S1: Supporting information. [file EMI4-11-206-s001.doc]

**Experimental Procedures**

**Sample collection**

We sampled oak, beech, spruce, larch and pine trees in three patches at different altitudes in August 2015 and July 2016 (Table S1). These patches are located in the Carnic Alps northeast of Italy, province of Udine. The temperature of Ampezzo (600 m altitude) was an average maximum of 28°C and a minimum of 23°C, while Passo Pura (1400 m altitude) was 25°C and 20°C with a mean annual precipitation was 816 mm. Soil samples surrounding oak, beech and spruce were collected from the first patch which was located 1.2 km from Ampezzo village, at an altitude of ~600 m. The second patch was an area of 1400 m altitude at the base of Mount Tinisia, 6 km from Ampezzo village. Samples from beech and spruce trees were collected as these trees were the most common species of this particular region, however, oak trees were absent. The third sampling patch was at a higher altitude of Mount Tinisia (1800 m and 1900 m). At an elevation of 1800 m larch trees dominated the sampling area of the mountain; therefore, three individual trees were sampled, however, a single isolated pine tree was present at the highest altitudes sampled (1900 m). Three soil samples from each tree were collected aseptically from the base not greater than 1m in radius and placed in sterile bags to avoid cross-contamination between tree samples. Samples were stored at 4°C until processing.

**DNA extraction from soil and PCR of ITS rDNA region**

DNA from the soil surrounding trees of different altitudes (Table S1) was extracted using the PowerSoil® DNA Isolation Kit (MO-BIO Laboratories, CA) according to the manufacturer’s instructions. Extraction of DNA from 0.25g of soil was carried out for each of the triplicate soil samples of each tree. The DNA concentration was then quantified using NanoDrop 2000 Spectrophotometer (Thermo Scientific, USA). To determine the soil fungal communities, the ITS region of rDNA was amplified using primers ITS5 (5´GGAAGTAAAAGTCGTAACAAGG`3) and ITS4 (5´ TCCTCCGCTTATTGATATGC`3) (Figure S1). DNA extracts were amplified in triplicate PCR reactions then to be pooled. This method was pioneered by Schmidt and co-workers who showed a more exhaustive snapshot of the soil fungal community was obtained from triplicate PCR reactions compared to a single PCR amplification, thus overcoming possible PCR biases towards certain fungi . The reaction consisted of 2µl extracted DNA, 0.6µM of each primer, 10µl 5x MyTaq buffer, 0.5µl MyTaq DNA Polymerase (Bioline, UK) and up to 50 µl with sterile MilliQ water. The PCR was performed using the following conditions of 1 cycle at 94 °C for 30 s, 35 cycles: 92 °C for 30 s, 56 °C for 30 s, 72 °C for 1 min and a final extension at 72 °C for 5 min.

The PCR products were concentrated to a volume of 25µl using an Eppendorf Concentrator Plus (Eppendorf, Germany). To increase the chance of *Saccharomyces sensu stricto* species recovery, the total volume was run on a 1% agarose gels and a region corresponding to a typical size of *Saccharomyces* spp ITS (850bp) was extracted using the QIAquick gel extraction kit (Qiagen, Germany). The extracted triplicates were pooled to create a single biological replicate for each tree, thus three replicates for each tree.

**ITS1 Amplicons library construction and sequencing**

A Library of ITS1 to be sequenced was prepared by amplification of the region using primers ITS1 and ITS2 with two Illumina overhang adapter sequences: forward overhang (5´TCGTCGGCAGCGTCAGATGTGTATAAGAGACAG`3) and reverse overhang (5´GTCTCGTGGGCTCGGAGATGTGTATAAGAGACAG`3) (Figure S1). The PCR reaction consisted of 2µl DNA, 0.6µM of each primer, 25µl 2x KAPA HiFi HotStart Ready Mix (KAPA Biosystems) and completed the volume to 50 µl with sterile MilliQ water. The PCR conditions were the same as the amplification of the ITS region described above then purified using QIAquick PCR purification kit (Qiagen, Germany). In order to multiplex the samples, a second round of PCR was undertaken to introduce dual of indices and Illumina sequencing adapters using the 96 sample Nextera XT Index Kit with following manufacturer's protocol (Illumina, USA) (Figure S1). Briefly, each DNA sample was labelled with a different combination of indices using Nextera index 1 primers (N7XX) and index 2 primers (S5XX). Each of the PCR reaction contained 5 µl DNA, 5 µl of each index primer, 25µl 2x KAPA HiFi HotStart Ready Mix and final volume made up with MilliQ water. The amplification conditions were as follow: 1 cycle at 95°C for 3 min, 8 cycles of 95°C for 30 s 55°C for 30 s, 72°C for 30 s and a final extension time at 72°C for 5 min. The final library of 27 samples was purified using AMPure XP beads (Beckman Coulter, UK) and the double-stranded DNA was accurately quantified using a Qubit 2.0 fluorometer (Invitrogen, UK). Before pooling the libraries, DNA concentration was determined by a 2100 Bioanalyzer (Agilent Technologies, Inc., CA). The concentrated libraries were diluted to 4 nM, and 5 µl of each library were then pooled. The samples were sequenced by Illumina MiSeq with paired-end 250bp reads. The sequences are submitted in the European Nucleotide Archives (ENA) database with accession number PRJEB27888.

**Processing of sequencing data**

The de-multiplexed raw FASTQ data was filtered by trimming barcodes, primers and low quality reads by Trimmomatic v0.36 . The read-pairs were then merged and chimeric sequences were identified and removed by Usearch v8.1.1861 . High-quality merged reads were assigned to taxa using the blast protocol in the QIIME 1.9.1 package with a cutoff at 0.001 against the 12_11 QIIME/UNITE ITS database (downloaded, April 2016) (<http://qiime.org/home_static/dataFiles.html>) in addition to all yeast ITS sequences extracted from NCBI GenBank database. [Sequences not assigned to taxa were clustered with Usearch protocol then further clustered into operational taxonomic units (OTUs). OTUs occurring with a single sequence in one sample (singletons) were removed from the dataset at this stage of the analysis, as they may be PCR or sequencing artefacts. To construct rarefaction curves, we used the QIIME package.](#_ENREF_1)  [Sequences with no match to the reference database](#_ENREF_2) were assigned as ‘No blast hit’. The identity of *Saccharomyces* species sequences was confirmed by alignment with the ITS1 region of different *Saccharomyces* populations using MEGA7  [and individually compared to reference species in the NCBI database](#_ENREF_4) . With exception of *S. uvarum* and *S. eubayanus*, all other pairs of species in the *sensu stricto* group differ by at least two nucleotides in the ITS1 region. The error rate for our Taq Polymerase procedure is ~1.1 x 10^-4^ , but there are three possible errors at any locus, and only one of those errors will lead to a particular misidentification. There are 7 different ways that any particular read can be misidentified (one for each incorrect species). So, for species pairs differing by two nucleotides in the ITS1 region (which is conservative), the probability of misidentifying any given read is 6 x (1.1 x 10^-4^)/3)^2^ = 9.4 x 10^-9^. Our data set includes 1387 *Saccharomyces* reads, so the probability that we made even one misidentification anywhere in our data set (with the exception of identifying *S. uvarum* as *S. eubayanus* or vice versa) is 1.1 x 10^-5^. *S. eubayanus* and *S. uvarum* differ by a single nucleotide. The base pair change going from *S. uvarum* to *S. eubayanus* is C->T, and C->T error rate during the Taq Polymerase procedure is approximately 7.3 x 10^-5^ . Our data set includes 14 reads of *S. uvarum* and 3 reads of *S. eubayanus*. The 14 *S. uvarum* reads came from 3 different samples (*i.e.* 3 different amplification cycles), and the 3 *S. eubayanus* reads came from 2 different samples (at least 3 independent *S. uvarum* and 2 independent *S. eubayanus* reads). The probability of making at least the minimum number of misidentifications (*i.e.* ≥2, for the number of independent samples in which we recorded *S. eubayanus*) in the maximum possible number of opportunities (*i.e.* 16 total for the 14 *S. uvarum* reads plus the 2 *S. eubayanus* samples that might be misidentified *S. uvarum*) was calculated using a binomial test for the probability of at least 2 events in 16 opportunities when the probability of each event is 7.3 x 10^-5^. This yields a result of 6.4 x 10^-7^, which is the probability that *S. eubayanus* was misidentified for *S. uvarum*.

**Sequences alignment**

ITS1 Sequences of *Saccharomyces* species were retrieved from the Genbank database , EMBL database and SGRP (*Saccharomyces* Genome Resequencing Project). MUSCLE tool was used to construct a multiple sequence alignment of mycobiome sequences with *Saccharomyces* species of different populations .

**Statistical analysis**

Two of the patches we sampled included both oak and beech trees. We calculated Jaccard similarity coefficients for fungal communities i) near different tree species in the same patch, and ii) near the same tree species in different patches. Comparing these coefficients offer a first pass at understanding whether the patch or the host tree has a great effect on community composition at the spatial scale of our study.

We modelled read counts as a function of species, patch and the interaction between species and patch using Poisson regressions implemented in the R package lme4 . To account for correlation in counts from the same tree species, the same tree and the same sample, we included random effects of trees species, individual tree and sample in our models. We selected the best model that included all or a subset of our fixed-effect predictors using Akaike’s Information Criterion . To estimate p-values for pairwise differences i) among individual species abundance across all patches, and ii) among patches in total *Saccharomyces* abundance, we used a fully non-parametric (*i.e*., “cases”) bootstrapping approach . That is, we resampled trees within patches and samples within trees, and then resampled each species count from a Poisson distribution with an expectation equal to the observed value. We repeated this process to create 10^4^ simulated data sets. We fit models to each simulated data set and used the distributions of effect sizes in the simulated data sets to estimate the bias-corrected and accelerated confidence intervals around the observed effect sizes of each predictor . We controlled the study-wise error rate for multiple comparisons using the Holm-Šidák approach . To confirm these results, we also applied the Holm-Šidák approach using p-values obtained from Wald tests and likelihood ratio tests. Because the number of potential interactions between species and patches in our model was large, and because the number of samples per species per patch was small, we did not attempt pairwise comparisons among interaction strengths.

**Supplementary Information**

**
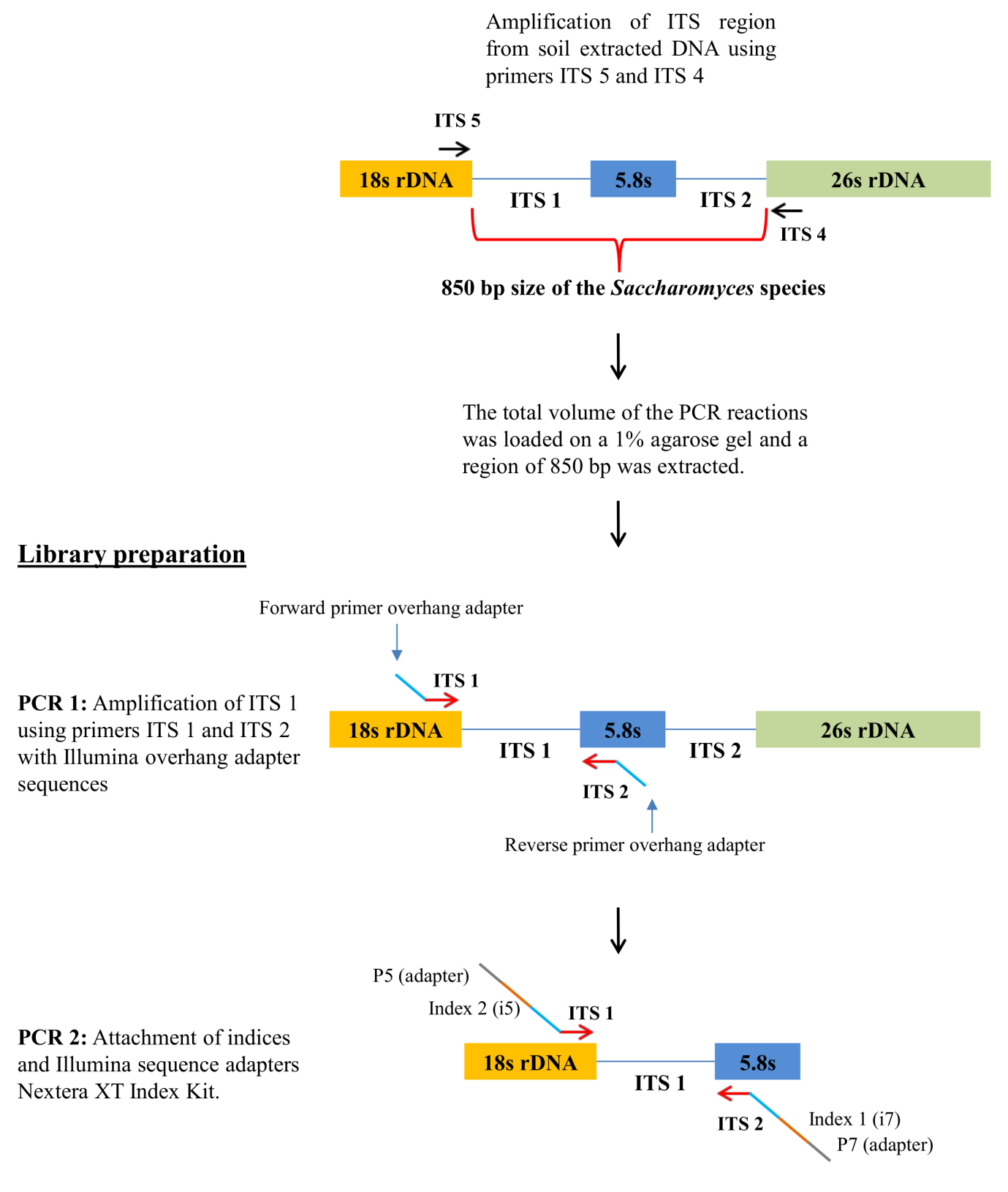
**

**Figure S1. Protocol for ITS amplification from soil DNA and ITS1 Illumina MiSeq library preparation.**


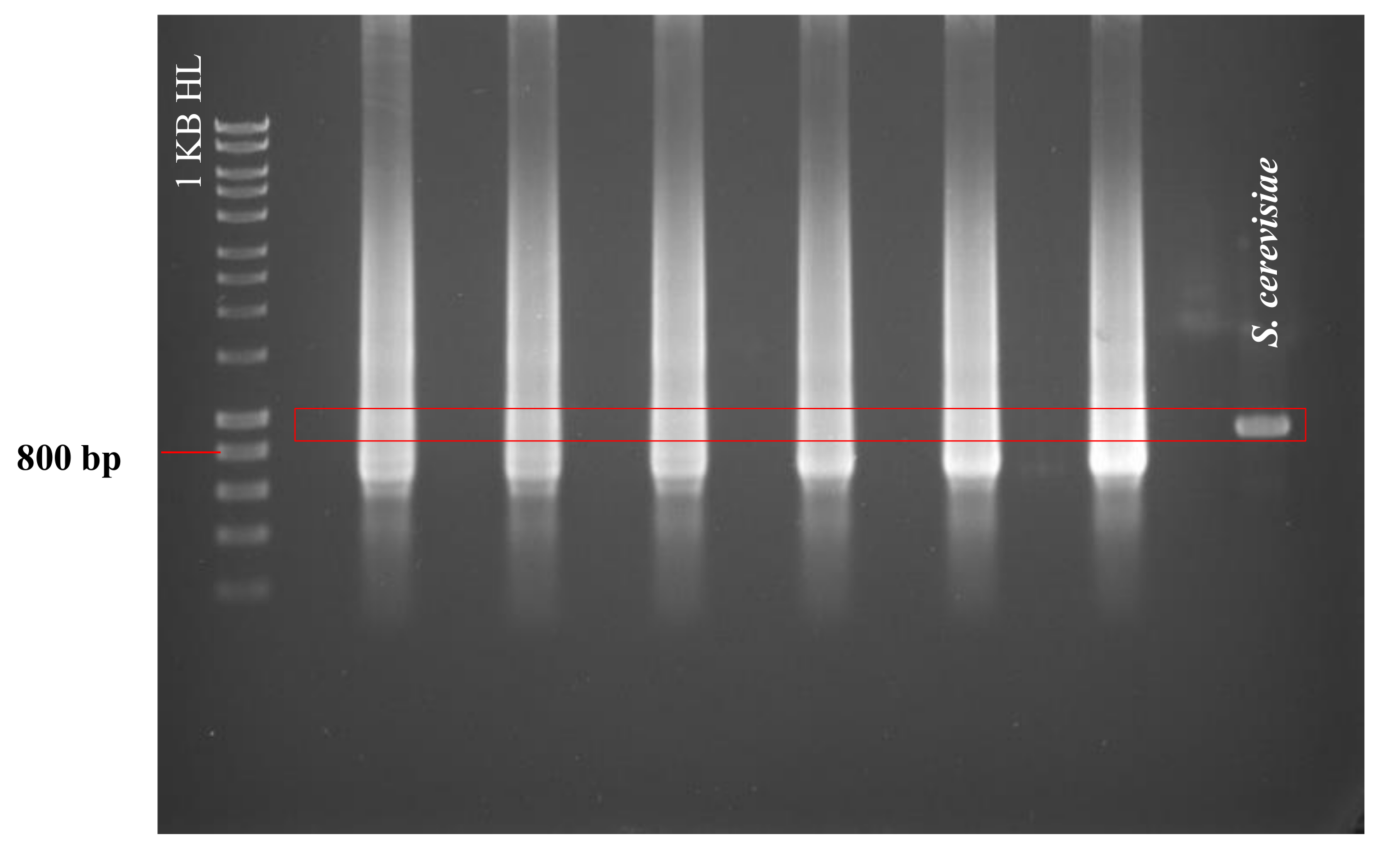


**Figure S2**. **An example of the 850 bp ITS1-5.8s-ITS2 region extracted from the agarose gel.** Each PCR reaction replicate was treated individually by concentrating the PCR product and loading the total amount on a 1% agarose gel. Under a UV illuminator, DNA bands equivalent to approximately 850 bp were extracted in comparison with *S. cerevisiae* as a control. Extraction of the required band was evaluated by the 1 Kb hyper ladder (Bio lab, UK).

**
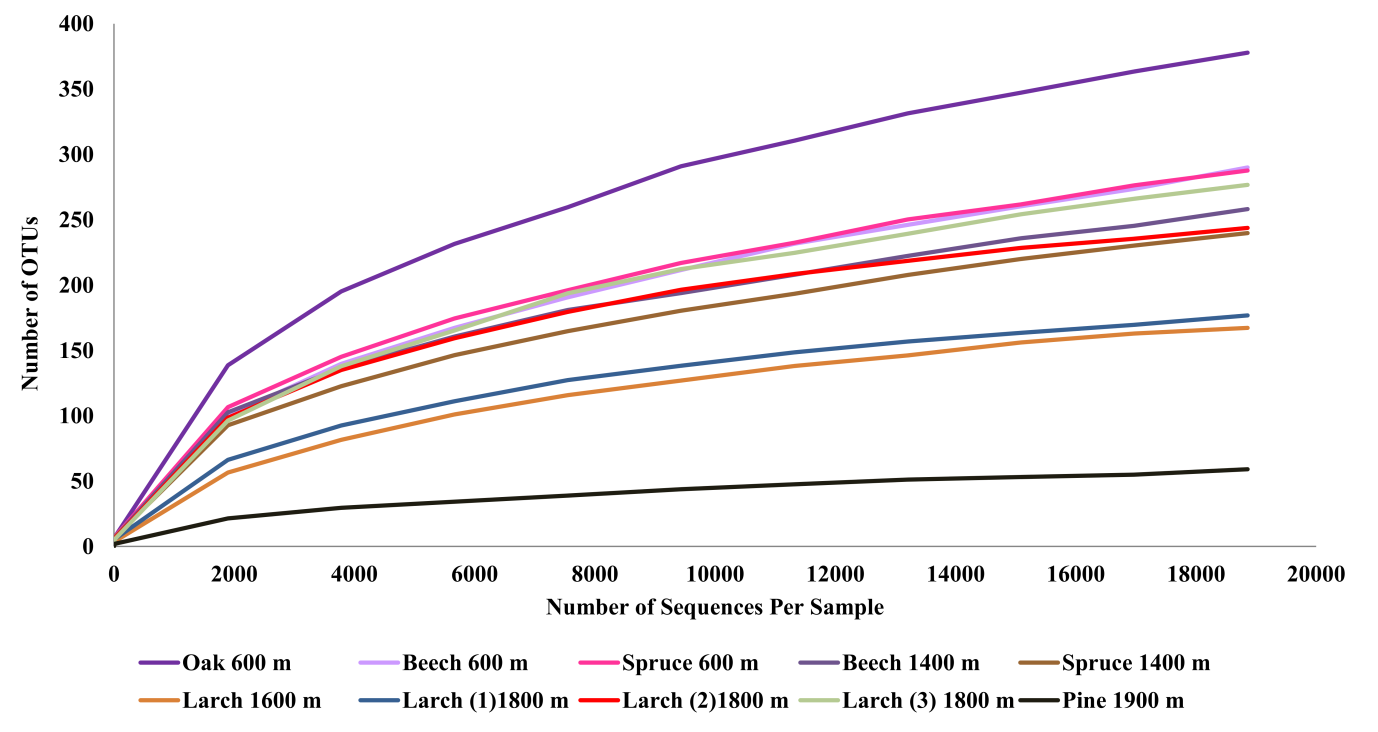
**

**Figure S3**. **Rarefaction analysis of species richness in each soil sample based on 99% OTU clustering.**

**
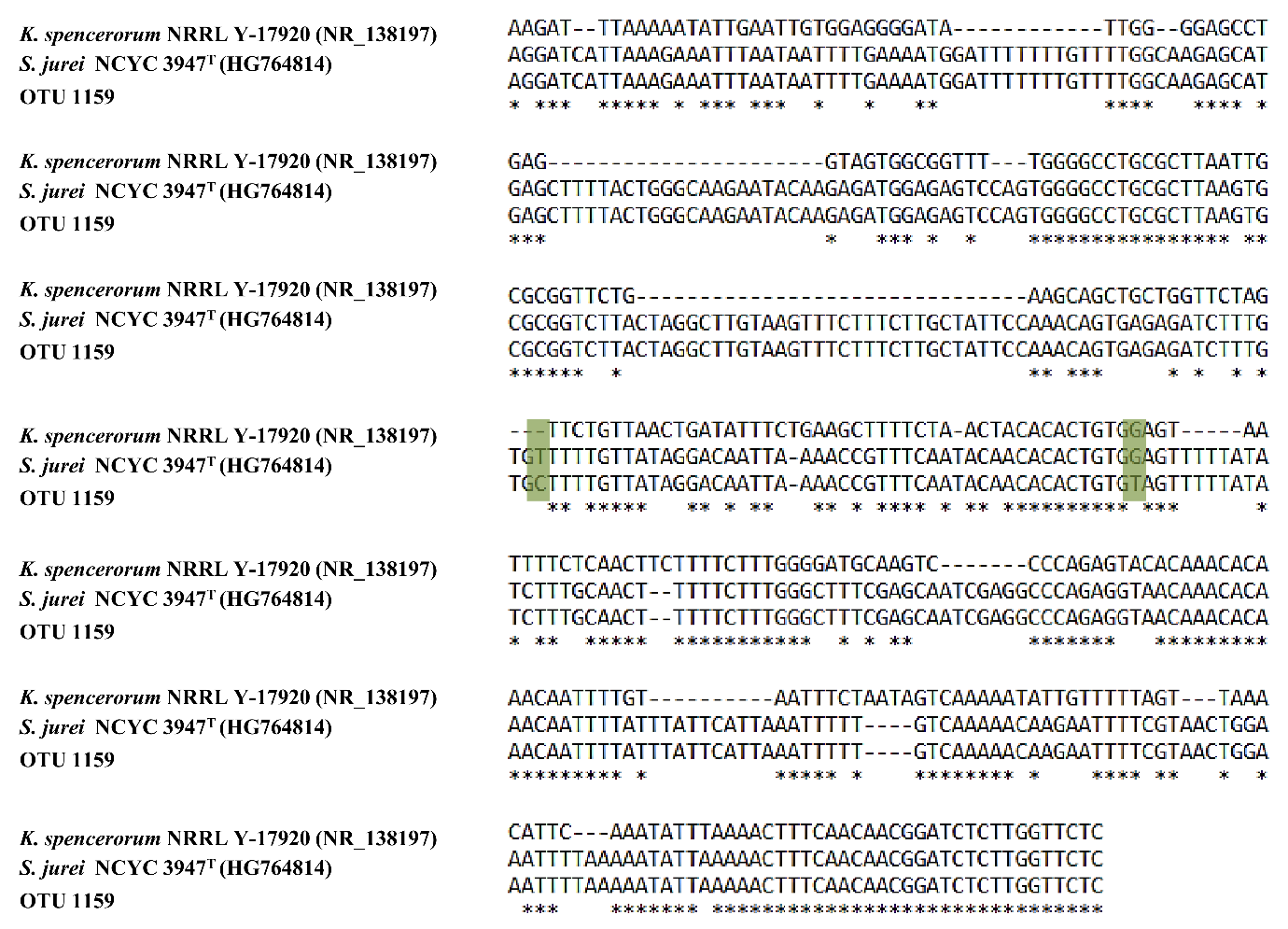
**

**Figure S4. *S. jurei* OTU 1159 alignment with ITS1 of *S. jurei* NCYC 3947^T^ and *K.* *spencerorum* NRRL Y-17920.** The green shaded regions indicate the nucleotide differences between OTU 1159 and *S. jurei* NCYC 3947^T^. The strains GenBank accession numbers are in parentheses.


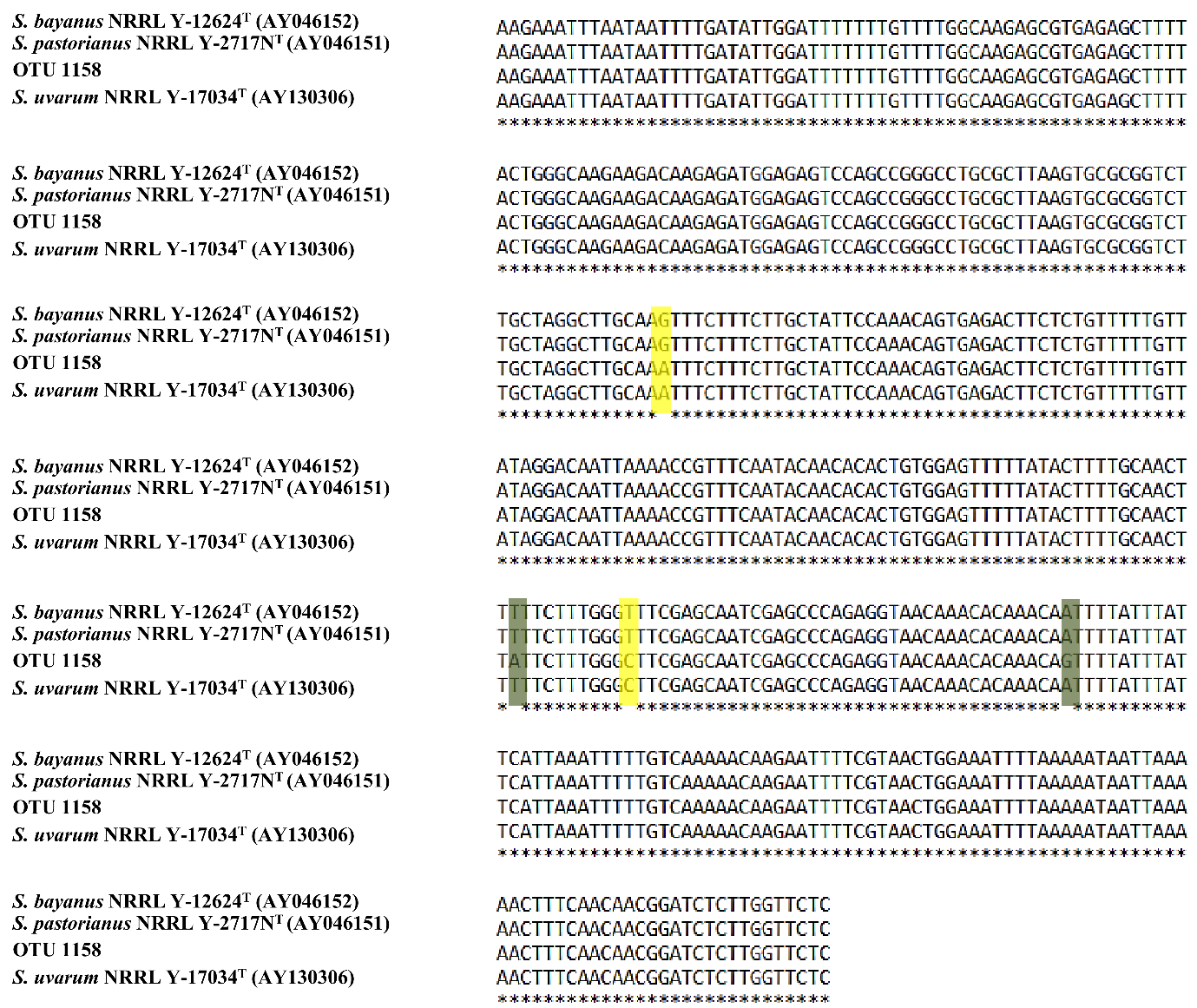


**Figure S5. *S. uvarum* OTU 1158 alignment with ITS1 of *S. bayanus* NRRL Y-12624^T^, *S. pastorianus* NRRL Y-2717N^T^ and *S. uvarum* NRRL Y-17034^T^.** The green shaded regions show base pair substitutions between OTU 1158 and the strains in the alignment. Yellow regions indicate the specific similarity between OTU 1158 and *S. uvarum* NRRL Y-17034^T^, thus differentiating the strains from *S. bayanus* NRRL Y-12624^T^ and *S. pastorianus* NRRL Y-2717N^T^. The strains GenBank accession numbers are in parentheses.

**
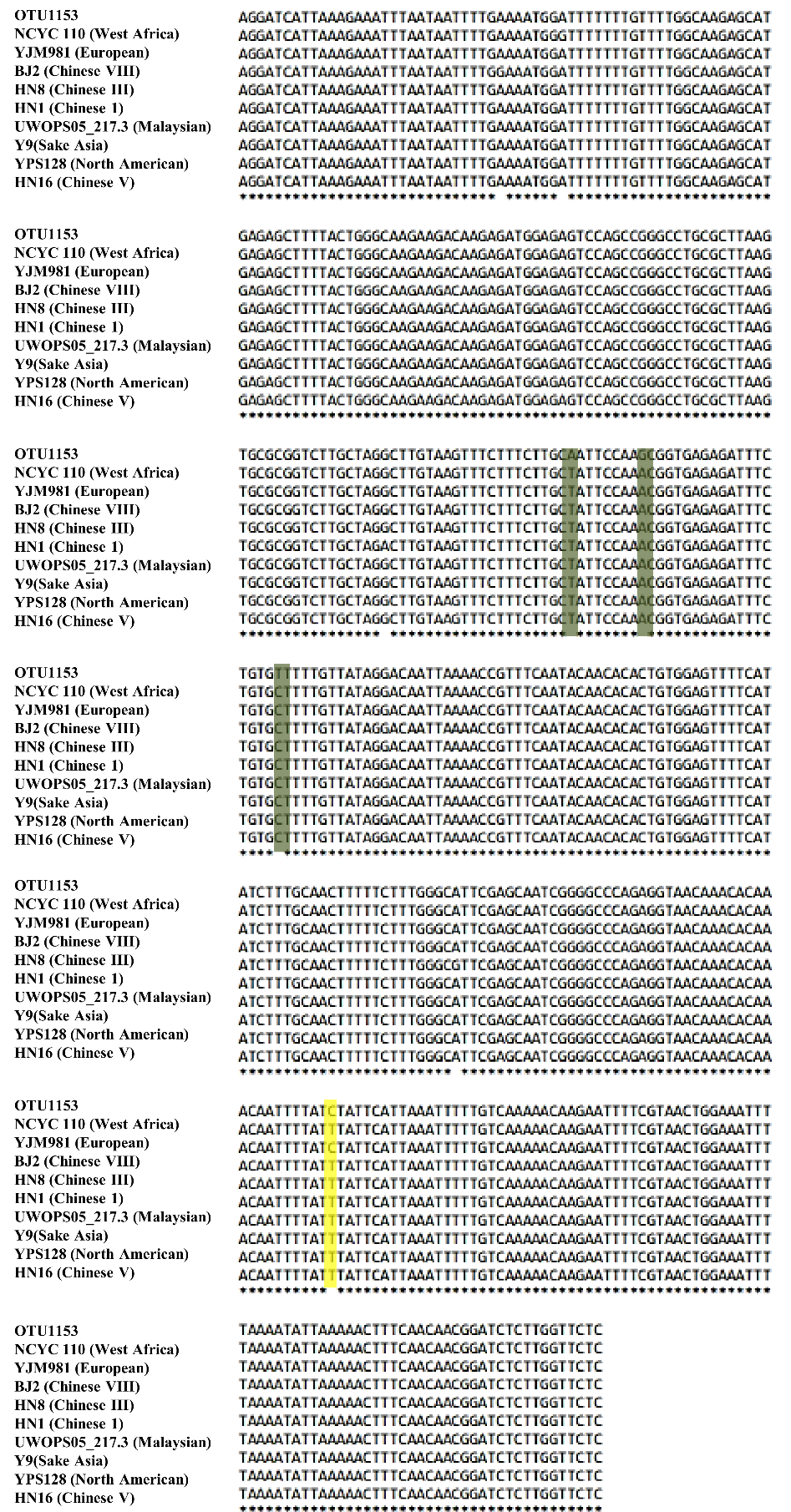
**

**Figure S6. *S. cerevisiae* OTU 1153 sequence alignment with ITS1 of *S. cerevisiae* strains representatives of different populations.** Strains with variation in ITS1 sequence within the Chinese population were chosen for the alignment. The green shaded nucleotides indicate the base pair differentiating *S. cerevisiae* OTU 1153 from the strains in the comparison. The yellow region highlights the nucleotide shared between OTU 1153 and the European strain.

**
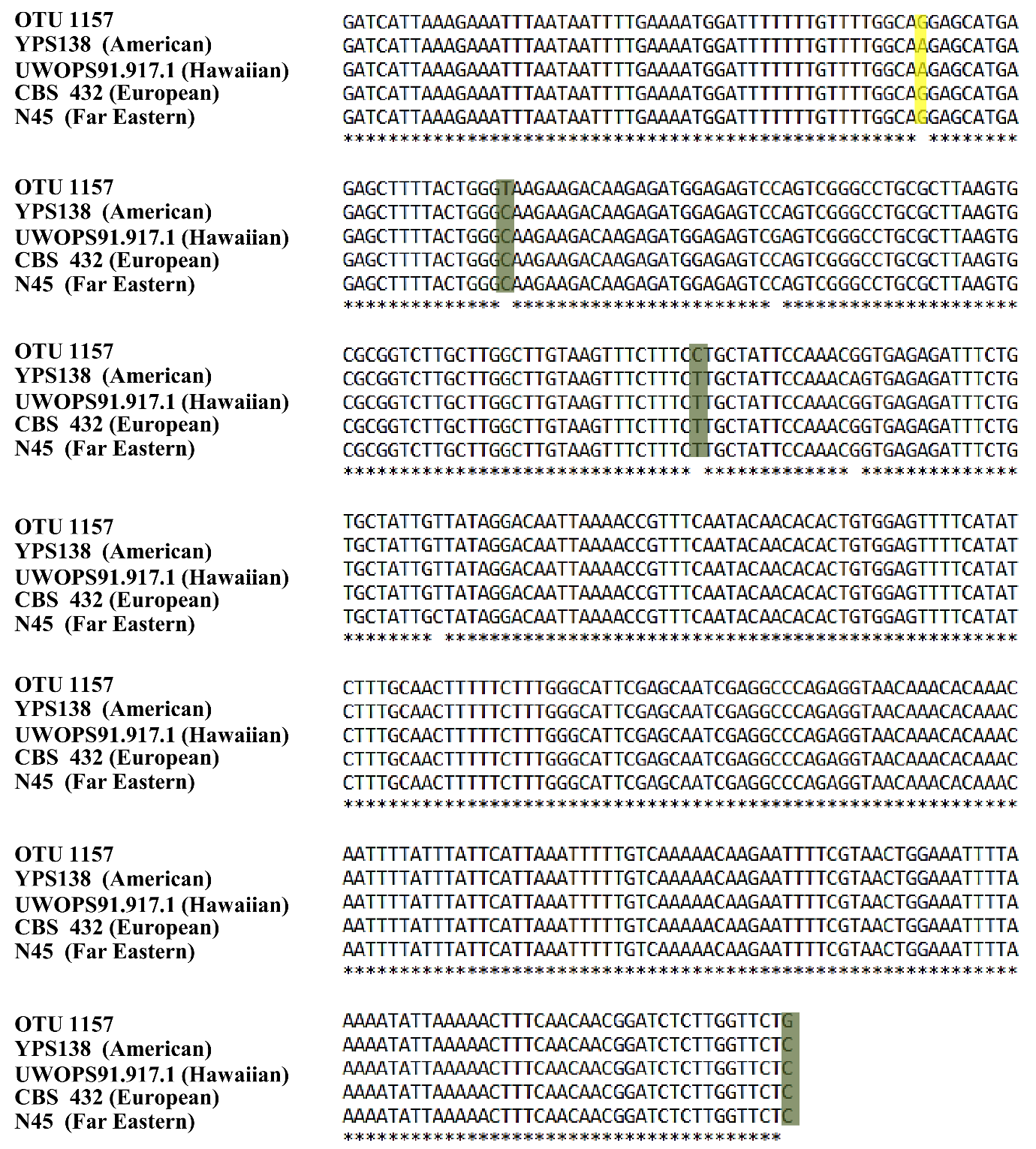
**

**Figure S7. *S. paradoxus* OTU 1157 sequence alignment with strains representing European, American, Hawaiian and Far Eastern populations.** The green areas show the base pair unique to *S. paradoxus* OTU 1157. The yellow shaded area highlights the shared base pair nucleotides between OTU 1157, European and Far Eastern *S. paradoxus* strains.

**
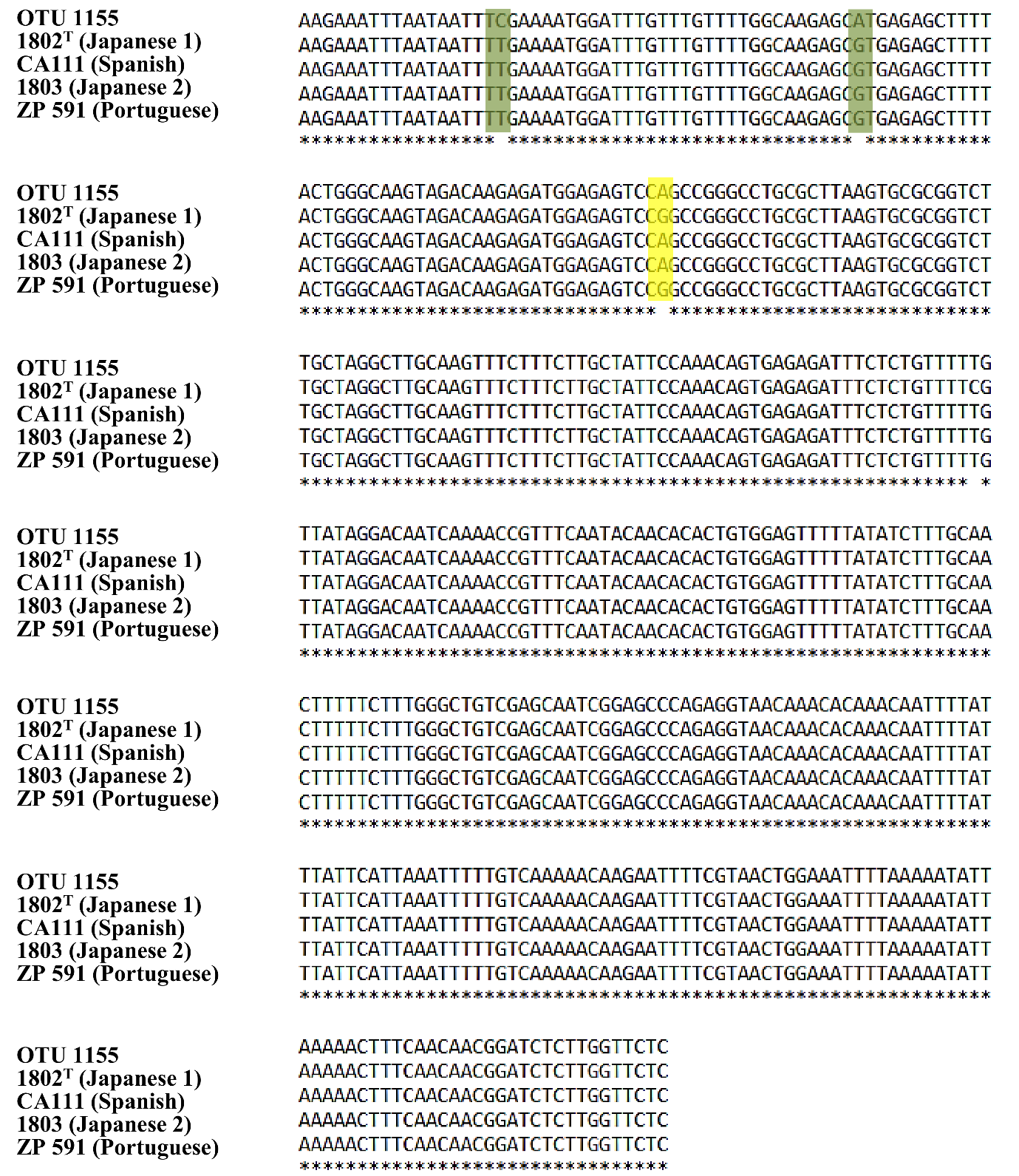
**

**Figure S8. *S. kudriavzevii* OTU 1155 sequence alignment with ITS1 of *S. kudriavzevii* strains representing European and Asian population**. The yellow shaded area indicate the location of the base pair difference between the European strains, including the sequence obtained in this study, and the Asian strains. The green shaded nucleotides show the region that differentiates OTU 1155 sequence from the other *S. kudriavzevii* strains.

**
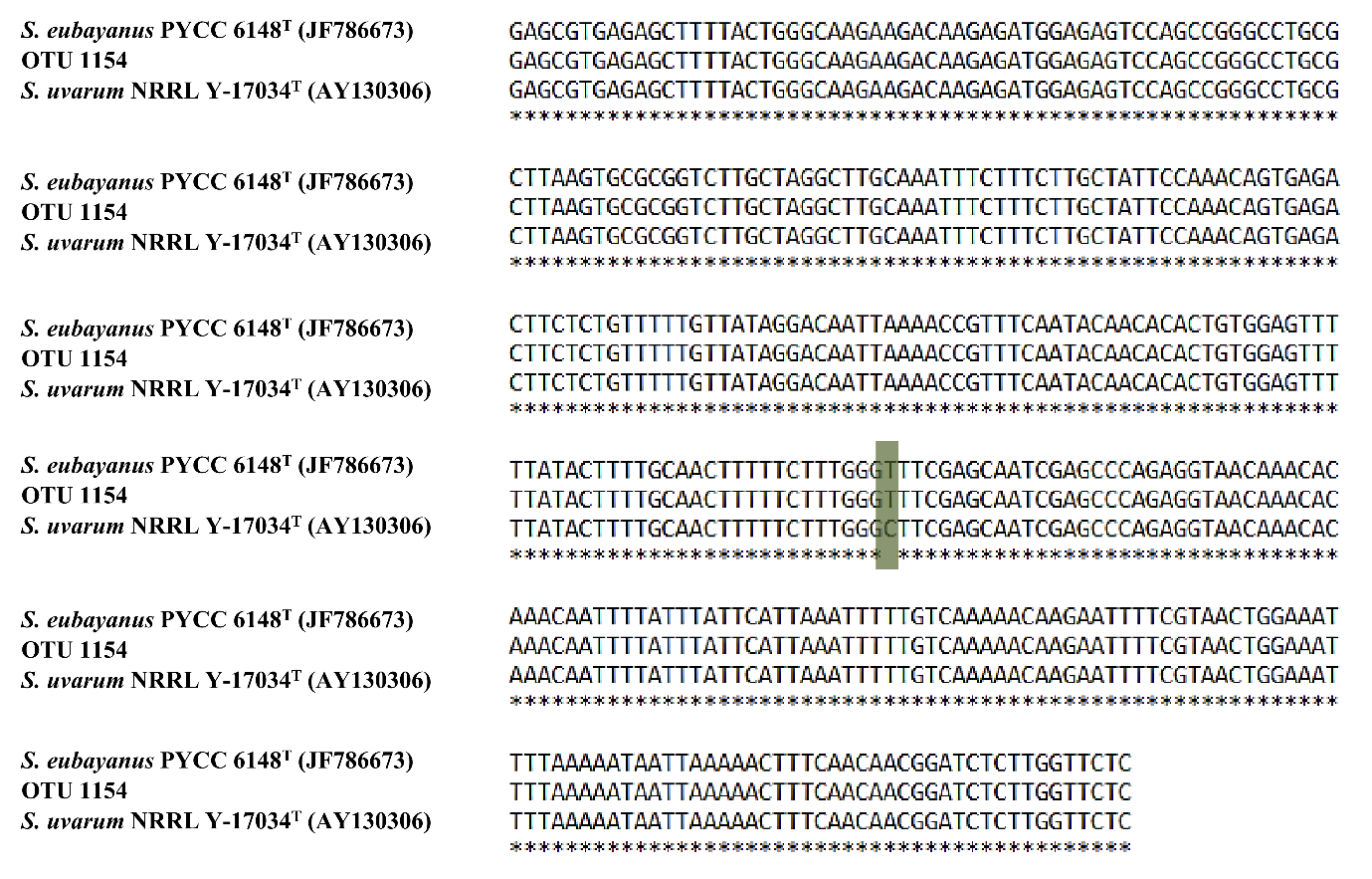
**

**Figure S9. *S. eubayanus* OTU 1154 sequence alignment with *S. eubayanus* PYCC 6148^T^ and *S. uvarum* NRRL Y-17034^T^**. The shaded area highlights the base pair that differentiates *S. eubayanus* OTU 1154 and *S. eubayanus* PYCC 6148^T^ from *S. uvarum* NRRL Y-17034^T^. The strains GenBank accession numbers are in parentheses.

**References**

Bates, D., Mächler, M., Bolker, B., and Walker, S. (2015) Fitting Linear Mixed-Effects Models Using lme4. *2015* **67**: 48.

Benson, D.A., Cavanaugh, M., Clark, K., Karsch-Mizrachi, I., Lipman, D.J., Ostell, J., and Sayers, E.W. (2013) GenBank. *Nucleic Acids Res* **41**: D36-42.

Bolger, A.M., Lohse, M., and Usadel, B. (2014) Trimmomatic: a flexible trimmer for Illumina sequence data. *Bioinformatics* **30**: 2114-2120.

Burnham, K.P., and Anderson, D.R. (eds) (2002) *Model Selection and Multimodel inference: a Pracical Information-Theoretic Approach*. New Tork: Springer-Verlag.

Caporaso, J.G., Kuczynski, J., Stombaugh, J., Bittinger, K., Bushman, F.D., Costello, E.K. et al. (2010) QIIME allows analysis of high-throughput community sequencing data. *Nat Methods* **7**: 335-336.

DiCiccio, T.J., and Efron, B. (1996) Bootstrap Confidence Intervals. *Statistical Science* **11**: 189-212.

Edgar, R.C. (2004) MUSCLE: multiple sequence alignment with high accuracy and high throughput. *Nucleic Acids Res* **32**: 1792-1797.

Edgar, R.C. (2010) Search and clustering orders of magnitude faster than BLAST. *Bioinformatics* **26**: 2460-2461.

Jaccard, P. (1912) The distribution of the flora in the alpine zone. *New Phytologist* **11**: 37-50.

Kumar, S., Stecher, G., and Tamura, K. (2016) MEGA7: Molecular Evolutionary Genetics Analysis Version 7.0 for Bigger Datasets. *Mol Biol Evol* **33**: 1870-1874.

Leeden, R.v.d., Meijer, E., and Busing, F.M.T.A. (2008) Resampling Multilevel Models. In *Handbook of Multilevel Analysis*. Leeuw, J.d., and Meijer, E. (eds). New York, NY: Springer New York, pp. 401-433.

Ryan, T.A. (1960) Significance tests for multiple comparison of proportions, variances, and other statistics. *Psychol Bull* **57**: 318-328.

Schmidt, P.-A., Bálint, M., Greshake, B., Bandow, C., Römbke, J., and Schmitt, I. (2013) Illumina metabarcoding of a soil fungal community. *Soil Biology and Biochemistry* **65**: 128-132.

Tindall, K.R., and Kunkel, T.A. (1988) Fidelity of DNA synthesis by the *Thermus aquaticus* DNA polymerase. *Biochemistry* **27**: 6008-6013.

White, T.J., Bruns, T., Lee, S., and Taylor, J. (1990) Amplification and Direct Sequencing of Fungal Ribosomal RNA Genes for Phylogenetics. In *PCR protocols: a guide for methods and applications*. M. A. Innis, D. H. Gelfand, Sninsky, J.J., and White, T.J. (eds): New York, pp. 315 - 322.
